# Novel Method of Cervicoplasty Using Autologous Peritoneum for Cervicovaginal Atresia

**DOI:** 10.1055/s-0040-1701213

**Published:** 2020-02-11

**Authors:** Kazunari Fujino, Yuko Ikemoto, Mari Kitade, Satoru Takeda

**Affiliations:** 1 Department of Obstetrics and Gynecology, Juntendo University Faculty of Medicine, Tokyo, Japan

**Keywords:** cervicovaginal atresia, cervical hypoplasia, vaginal agenesis, uterovaginal anastomosis, vaginoplasty, autologous peritoneum, laparoscopic surgery

## Abstract

Cervicovaginal atresia with a functional uterus is rare. There are no established surgical methods to treat this condition, and only a few reports have been published on surgical techniques. Furthermore, postoperative complications, such as restenosis, often require reoperation. A 19-year-old woman was pointed out cervical hypoplasia and referred to our hospital for further examination and treatment. A pelvic examination revealed that the vagina had a slight recession with a blind end. Transrectal ultrasound and pelvic magnetic resonance imaging revealed congenital vaginal agenesis and cervical hypoplasia. Elective surgery was performed after reshaping the vagina. A radical surgery was performed 10 months later after sufficient self-dilation by using Frank's technique in an outpatient setting. At first, we approached by laparoscopically to correct autologous peritoneum and to bladder detach, then the cervical canal was identified. Next, a skin biopsy punch was used several times to hollow out the cervical tissue to shape and expand the cervical canal. A catheter was then placed in the uterus and autologous peritoneum was wrapped around it and fixed to the cervical canal. The catheter was removed 6 weeks postoperatively, and the patient continued dilating her vagina until she was able to have sexual intercourse, and then stopped the self-dilation. Eight months postoperatively, the patient did not report any menstrual irregularities. It is important to make corrections to prevent restenosis of the vagina and cervical canal and prevent the symptoms from recurring. Make use of autologous peritoneum as graft onto the cervical canal is effective method for the treatment of cervicovaginal atresia.

Cervicovaginal atresia with a functional uterus is rare. This condition is caused by an abnormal formation or fusion of the Müllerian ducts and is often associated with vaginal aplasia. It may be diagnosed before or after puberty during medical examinations for periodic abdominal pain, amenorrhea, or dyspareunia. A total hysterectomy was the recommended treatment of choice for curative treatment, although conserving fertility required invasive vaginoplasty and cervicoplasty. However, identifying and reshaping the cervix during surgery can be difficult, and there is a high risk of damage to the bladder or intestines. In addition, a postoperative restenosis and closure of the vaginal or cervical canal can be a recurrence of the symptoms. Additionally, as many patients are young, minimally invasive surgeries that will not lead to recurrence are desirable. Recently, there have been a few reports of vaginal surgeries that combined laparoscopic or robot-guided techniques. We report a case in which we adjusted the pre- and postoperative management, and combined laparoscopic surgery with vaginal surgery using autologous peritoneum and a variety of surgical instruments to perform a minimally invasive and safe vaginoplasty and cervicoplasty with no restenosis or closure.

## Case Presentation

**Fig. 1:**
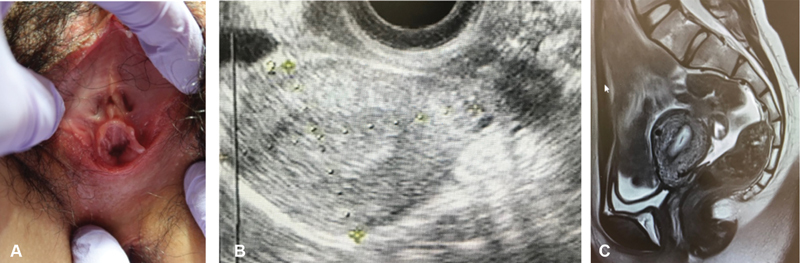
( **A** ) Vagina with blind end. ( **B, C** ) Uterine cervix is thin, restiform shaped, and the cervical canal line structure is indistinct. Bilateral adnexa are normal. Accumulation of menstrual blood is seen in the uterine body.

The patient was a 19-year-old nulligravida woman who was examined for primary amenorrhea at another hospital. On examination, the vulva was normal, but vaginal agenesis was observed; the uterine cervix was restiform shaped, and the cervical canal line structure was indistinct, which indicated cervical hypoplasia. The patient was referred to our hospital for further examination and treatment. A pelvic examination revealed that the vagina had a slight recession with a blind end. Transrectal ultrasonography showed absence of a cervical canal line in the uterine cervix, fluid accumulation in the intrauterine cavity, and normal ovaries on both sides. Pelvic magnetic resonance imaging showed similar findings ( Fig. 1 ). Chromosomal testing was 46 XX. Based on this, congenital vaginal agenesis and cervical hypoplasia were diagnosed and a decision was made to perform an elective surgery.

## Surgical Technique

**Fig. 2:**
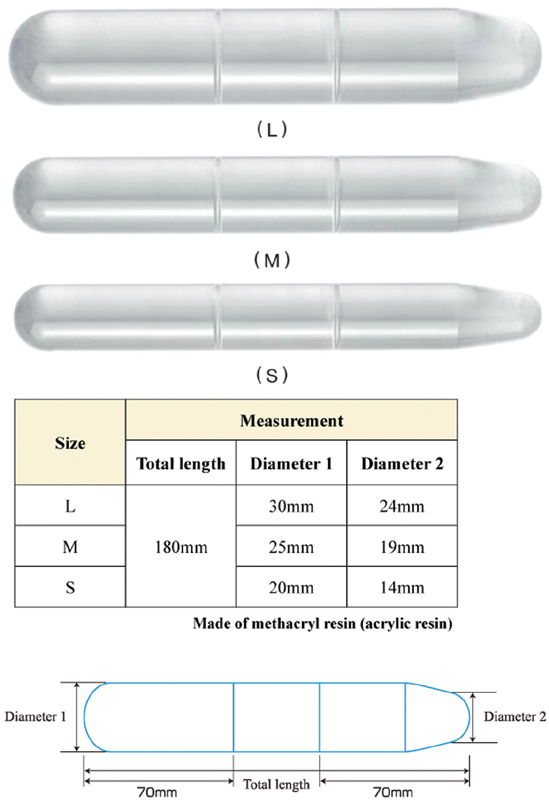
Vaginal dilators (Atom Medical) developed by Takeda are easy to hold and convenient for self-management. The point of the dilator is sharp and easy to care.

**Fig. 3:**
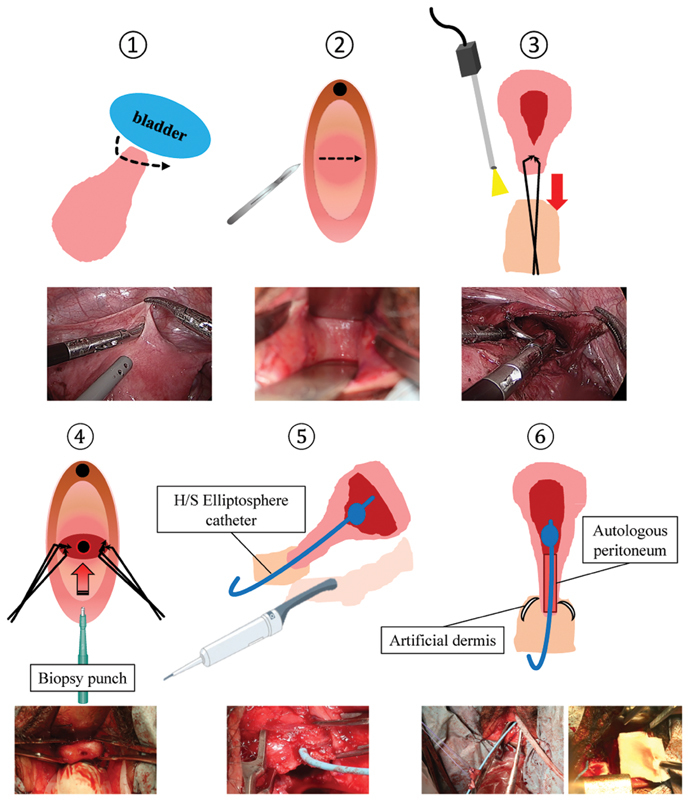
Schematic diagram of the surgery. (1) Laparoscopically, the hypoplastic uterine cervix was identified, then peritoneal peritonectomy using for grafted onto the cervical canal was performed. (2) In the vaginal surgery, incision was made into the blind end to shape the vagina. (3) Traction and fixation of the hypoplastic uterine cervix was performed from the vaginal side. (4) Skin biopsy punch of 3 mm was used to hollow out and resect the external cervical Os; communication between the cervical canal and dilated uterine cavity was created. (5) Catheter used in hysterosalpingography was placed in the uterine cavity guided by transrectal ultrasonography. (6) Autologous peritoneum was fixed to the catheter and placed in the cervical canal. Artificial dermis was used to promote epithelization and prevent infections in the vagina, which had damaged epithelium.

**Fig. 4:**
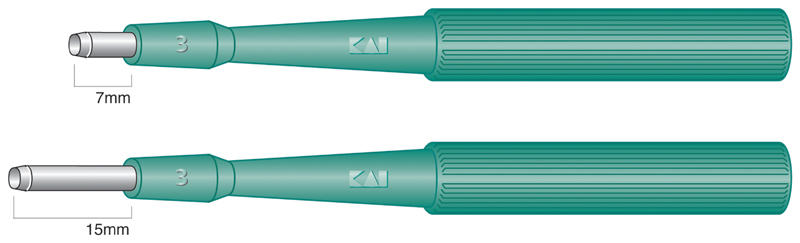
Biopsy punch (Kai Industries) is able to choose diameter and length of blade for each size of cervix.

**Fig. 5:**
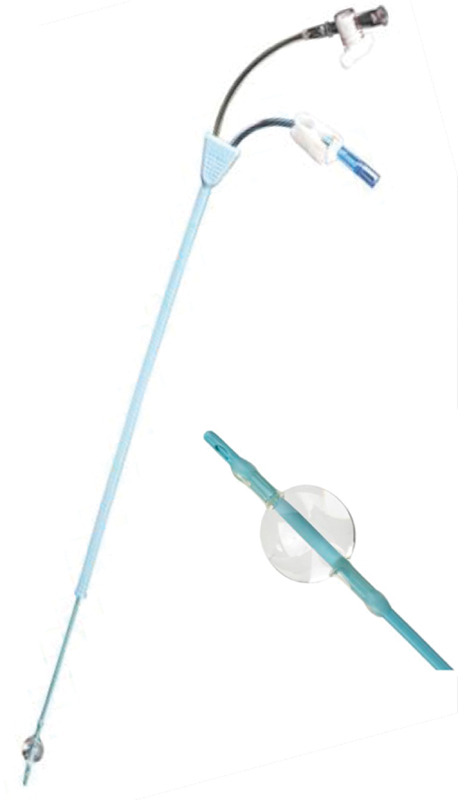
H/S Elliptosphere catheter (CooperSurgical) used for hysterosalpingography is suitable to place in uterus.

The management was divided into three steps: (1) preoperative management, (2) surgery, and (3) postoperative management. The procedure was as follows:

1. Preoperative management
Due to the presence of vaginal agenesis, the vagina was reshaped preoperatively in an outpatient setting. The patient was given instructions on the use of a vaginal Dilator S (Atom Medical Corp., Tokyo, Japan) ( Fig. 2 ) to be used at home (Frank's technique). After 10 months, she could accommodate a larger sized Dilator M and had a depth of 7 cm, which made sexual intercourse possible. Subsequently, it was decided to perform a radical surgery.
2. Surgery ( Fig. 3 )
  - Laparoscopic approach
    Peritoneum to be grafted onto the cervical canal was harvested laparoscopically. The peritoneum at the vesicouterine pouch was incised, and the bladder and uterine cervix were detached and developed to identify the hypoplastic uterine cervix.
  - Vaginal approach
    For the vaginal surgery, a cold knife was used to make a 1.5-cm incision in the blind-end of the vagina. Taking care not to damage the bladder or rectum, the laparoscopic light source was used as a landmark while reshaping the vagina by excavating toward the abdominal cavity.
  - Laparoscopic-assisted vaginal approach
    Under laparoscopic observation, traction and fixation of the uterine cervix inside the vagina were performed from the vaginal side.
  - Vaginal approach
To shape the cervical canal, a 3-mm skin biopsy punch (Kai Industries, Tokyo, Japan) was used to hollow out and resect the external cervical Os ( Fig. 4 ). Next, a communication between the shaped cervical canal and the dilated uterine cavity was created.
  - Vaginal approach
An H/S Elliptosphere catheter (CooperSurgical, CT, USA) used for hysterosalpingography was placed in the uterine cavity ( Fig. 5 ).
  - Vaginal approach
    The laparoscopically harvested autologous peritoneum was wrapped around the catheter and fixed in the cervical canal. Artificial dermis (Pelnac, Smith & Nephew) was used to promote epithelization and prevent infections in the vagina, which had damaged epithelium.
3. Postoperative management
  Two weeks postoperatively, epithelization of the vaginal wall was confirmed, and the patient was discharged from the hospital with the catheter in place. Six weeks postoperatively, the catheter was removed from the uterus and epithelization of the uterine and cervical cavities was reconfirmed. To prevent vaginal restenosis, self-dilation with a vaginal dilator M was resumed, which the patient had stopped when she had become capable of sexual intercourse.
  Two months postoperatively menstruation was confirmed, which was without any pain or difficulty in discharging the menstrual blood. Eight months postoperatively, restenosis has not occurred.

## Discussion

The female genitalia differentiate and develop from the Müllerian ducts and urogenital sinus during the embryonic stage. The former become the ovaries, uterine body, cervix, and upper third of the vagina, while the latter becomes the lower two-thirds of the vagina. Formation of the female internal genitalia consists of the following steps: (1) formation of two Müllerian ducts, (2) fusion of the Müllerian ducts, (3) fusion of the lower Müllerian ducts and urogenital sinus, and (4) absorption of the septum. Abnormal development at any of these stages can lead to morphological defects.

Vaginal hypoplasia is seen in 1 out of every 4,000 to 5,000 women, with the Rokitansky–Kuüster–Hauser syndrome being a well-known congenital vaginal defect. In cases of cervical hypoplasia or aplasia similar to the present one, the abnormality is thought to have occurred relatively early during the formation of the Müllerian ducts, although the etiology remains unclear. Cervicovaginal atresia is even rarer, with less than 200 cases reported from when it was first described by Roberts et al from 1942 to 2011. 1 Only 2 to 7% of cervicovaginal atresia cases have functional uteruses with vaginal agenesis. 2 The conventional treatment was to create a communication between the uterine body and vagina. However, Buttram and Gibbons noted that, as the cervical canal created in these operations are fistulas that do not secrete cervical mucus and can cause severe ascending infections and the patients remain infertile. Therefore, hysterectomy is not recommended. 3 Recently, surgeries that conserve the uterus out of consideration of fertility have been reported. Most of the reports were of laparotomies, 4 5 6 7 although in 2008 Fedele et al reported using a laparoscope in a minimally invasive surgery with excellent cosmetic results. 8 Robot-assisted surgeries have also been reported. 9 In the present case, we combined laparoscopic surgery with vaginal surgery. The surgery was performed laparoscopically because it allowed us to observe the inside of the abdominal cavity and later harvest the free peritoneum. Further, laparoscopically developing the peritoneum at the vesicouterine pouch to confirm the uterine cervix and use of the laparoscope's light source as a landmark to secure the uterine cervix transvaginally was useful for avoiding complications and shortening the operation. There are few published reports on cervicoplasty techniques. Most have described creating a communication with the vagina by inserting a transuterine catheter via the uterine body and excavating sharply toward the uterine body from the vaginal blind end. However, a high risk of bladder or rectal damage from proceeding in a mistaken direction and the difficultly of the manipulations make this a difficult step. Particular care is needed when there is considerable distance between the uterus and the vaginal blind end. In the present case, domestic use of Frank's technique as a form of preoperative therapy shortened this distance and made the vaginal surgery safer. It is the American College of Obstetricians and Gynecologists committee's recommendation to use Frank's technique as the first-line approach in cases of vaginal aplasia. 10 At our hospital, we have found that dilators are easy to use at home, and those developed by Takeda have improved handles and tips and can also be used to prevent postoperative stenosis. 11

The most important things to note in the present case are the creation of a wide cervical canal and the efforts to achieve early epithelization of the cervical mucosa. A canal with a certain width needs to be created in cases of hypoplasia in which the cervical canal line is not visible, to allow menstrual blood to discharge sufficiently. In the present case, we adopted a skin biopsy punch to shape the uterine cervix ( Fig. 4 ). Biopsy punches are often used in dermatology and can cut out tube-like holes in tissue. Blades with different diameters and lengths can be selected to suit the design of the cervical canal. Others have reported inserting a Foley's catheter from the uterus to the vagina to prevent natural closing of the hollowed-out cervical area, obtaining good results with no restenosis. 9 In the present study, we used an H/S Elliptosphere catheter used in hysterosalpingography, which was passed from the vagina though the cervical canal to the uterine cavity. In addition, we promoted epithelization of the cervical canal mucosa by wrapping the autologous peritoneum collected laparoscopically around the catheter and fixing it in the uterine cervix. This vaginoplasty method was reported by Takeda 12 and is safer and cheaper than Gore-Tex 13 and artificial dermis. 7 14 The autologous peritoneum grafted onto the vagina had changed into squamous epithelium after 3 weeks, and after 8 weeks the patient was able to engage in sexual intercourse. This indicates that because autologous peritoneum changes the epithelium around where it is fixed, the peritoneum grafted onto the cervical canal will epithelize quickly without leaving a scar, which creates a cervical canal that does not restenose or close after removing the catheter from the uterus.

As discussed, the skillful combination of preoperative management, modifications to the surgical technique, device selection, early epithelization of the cervical canal mucosa through peritoneal grafting, and postoperative management were able to create a cervical canal that did not undergo restenosis or close. Later, care regarding pregnancy and childbirth will be necessary, which will require long-term follow-up. Furthermore, this surgical technique may be applicable in conditions such as double uterus or unilateral cervical canal hypoplasia.

## Conclusion

We developed a novel method of cervicoplasty that did not lead to restenosis or closure. A skin biopsy device was used to hollow out the cervical tissue and create a wide cervical canal to prevent restenosis. In addition, grafting autologous peritoneum onto the cervical canal was important in achieving early epithelization of the cervical mucosa.
